# Shear-Wave Elastography for the Differential Diagnosis of Breast Papillary Lesions

**DOI:** 10.1371/journal.pone.0167118

**Published:** 2016-11-28

**Authors:** Jin Chung, Won Kyung Lee, Eun-Suk Cha, Jee Eun Lee, Jeoung Hyun Kim, Young Hoon Ryu

**Affiliations:** 1 Department of Radiology, Ewha Womans University, College of Medicine, Seoul, Republic of Korea; 2 Department of Radiology, Yonsei University, College of Medicine, Seoul, Republic of Korea; 3 Department of Nuclear Medicine, Yonsei University, College of Medicine, Seoul, Republic of Korea; Semmelweis Egyetem, HUNGARY

## Abstract

**Objective:**

To evaluate the diagnostic performance of shear-wave elastography (SWE) for the differential diagnosis of breast papillary lesions.

**Methods:**

This study was an institutional review board-approved retrospective study, with a waiver of informed consent. A total of 79 breast papillary lesions in 71 consecutive women underwent ultrasound and SWE prior to biopsy. Ultrasound features and quantitative SWE parameters were recorded for each lesion. All lesions were surgically excised or excised using an ultrasound-guided vacuum-assisted method. The diagnostic performances of the quantitative SWE parameters were compared using the area under the receiver operating characteristic curve (AUC).

**Results:**

Of the 79 lesions, six (7.6%) were malignant and 12 (15.2%) were atypical. Orientation, margin, and the final BI-RADS ultrasound assessments were significantly different for the papillary lesions (*p* < 0.05). All qualitative SWE parameters were significantly different (*p* < 0.05). The AUC values for SWE parameters of benign and atypical or malignant papillary lesions ranged from 0.707 to 0.757 (sensitivity, 44.4–94.4%; specificity, 42.6–88.5%). The maximum elasticity and the mean elasticity showed the highest AUC (0.757) to differentiate papillary lesions.

**Conclusion:**

SWE provides additional information for the differential diagnosis of breast papillary lesions. Quantitative SWE features were helpful to differentiate breast papillary lesions.

## Introduction

Papillary lesions of the breast have a wide spectrum, including benign papilloma, atypical papilloma, and papillary carcinoma [1]. Managing patients with breast papillary lesions diagnosed by core needle biopsy (CNB) has been controversial. Previous studies [2, 3] have reported that surgical excision is recommended for an accurate diagnosis of benign papilloma diagnosed using ultrasound (US)-guided CNB. In contrast, some studies have reported that US-guided vacuum-assisted excision (VAE) is accurate and could be an alternative to surgery for benign papillary lesions [4–7]. Sohn et al. [4] reported no malignancies after excising benign papilloma diagnosed by US-guided CNB. Ko et al. [5] suggested that well-selected benign papilloma (category 3 or 4A papillary lesions < 1.5 cm) can be treated with US-guided VAE rather than surgical excision. Although management of breast papillary lesions is controversial, the current trend is to use US-guided VAE for well-selected benign papillary lesions diagnosed by US-guided CNB [4–7]. In contrast, papillary lesions with atypia should be treated by surgical excision [6, 7]. Therefore, sub-analysis of breast papillary lesions is essential for managing patients.

Previous US studies have revealed that nonparallel orientation, echogenic halo, posterior enhancement, mixed hyper- and hypoechoic or complex cystic echogenicity, and an uncircumscribed margin were associated with malignant papillary lesions [3, 8]. However, papillary lesions are difficult to diagnose as benign or malignant based on radiological findings, due to overlapping imaging findings [9, 10]. Previous studies have reported that elastography is useful for differentiating benign from malignant breast masses [11–16]. Choi et al. [17] reported that strain elastography improves the specificity of conventional US for differentiating breast papillary lesions. However, those studies used strain elastography, which applies manual compression, and the amount of breast lesion deformation relative to the surrounding normal tissue is measured and displayed as a color. Thus, data acquisition with strain elastography is operator dependent and significant inter-observer variability has been reported [15,16,18–20].

Shear-wave elastography (SWE) is based on local measurements of shear-wave propagation speed. SWE is less-operator dependent and highly reproducible [19–22]. SWE improves diagnostic performance when differentially diagnosing breast masses, compared to that of conventional US alone [21–26]. SWE can also be used to measure various quantitative and qualitative factors. However, the diagnostic performance of SWE for breast papillary lesions has not been investigated. Therefore, the purpose of this study was to evaluate the diagnostic performance of SWE for the differential diagnosis of breast papillary lesions.

## Materials and Methods

### Patients and Lesions

This retrospective study was conducted with institutional review board approval of Ewha Womans University Mokdong Hospital and a waiver of patient informed consent from the participants. All patient records/information was anonymized and de-identified prior to analysis. However, written informed consent was obtained from all patients for US-guided CNB and US-guided VAE or surgical excision prior to each procedure as a daily practice.

A total of 3074 US-guided breast CNBs had been performed at our institution, from January 2013 to January 2015. Of these, 217 (7.1%) were diagnosed as papillary lesions. All lesions underwent conventional US, prior to the CNB. We excluded lesions without a SWE image. We also excluded lesions that were not excised surgically or by US-guided VAE. We finally included 79 lesions in 71 women (mean age, 48 years; range, 21–85 years). The clinical features were reviewed retrospectively from the patient’s medical records and radiological reports. Breast density was recorded with mammographic reports. One of the breast radiologists randomly and independently reviewed and assessed the mammographic findings and BI-RADS final assessments were determined [27]. According to BI-RADS mammography lexicon, breast density was evaluated; breast composition A (The breasts are almost entirely fatty), breast composition B (There are scattered areas of fibrograndular density), breast composition C (Heterogeneously dense, which may obscure masses), and breast composition D (Extremely dense, which lowers sensitivity). Breast composition C and D were considered as dense breasts.

### Conventional US Data Acquisition and Analysis

Ultrasound was performed by one of four board certified radiologists (E.S.C., J.E.L., J.H.K., or J.C.) with 5–25 years of experience with breast imaging. Ultrasound unit with a 7.5-15MHz linear-array transducer (Aixplorer system, Supersonic Imagine, Aix en Provence, France) was used for conventional US and SWE images. Bilateral whole breast hand-held US examinations were performed by the radiologists.

One of the breast radiologists randomly and independently reviewed and assessed the conventional US images. All radiologists were aware of the clinical and mammographic features. The following data had been measured and recorded by the radiologist who had performed the US: lesion size (maximum diameter on B-mode US image), peripheral location (> 3cm from the nipple to the margin closest to the nipple), breast thickness (maximum vertical distance from the skin to the pectoralis muscle), lesion depth (vertical diameter from the skin to center of the lesion), and ductal dilatation (adjacent or connected ductal dilatation) [22, 28–30].

Conventional US findings were analyzed according to the BI-RADS lexicon, and BI-RADS final assessments were determined [27]. Shape (oval, round, or irregular), orientation (parallel or non-parallel to the skin), margin (circumscribed or not), echo pattern (hypoechoic, isoechoic, or complex cystic and solid), posterior features (absent or present), and vascularity (absent or present) were recorded. Dominant vascularity of the lesion was also determined as rim or internal vascularity. Final BI-RADS US assessments were determined for each lesion. BI-RADS category 4 was subdivided into 4A (low suspicion for malignancy), 4B (moderate suspicion for malignancy) and 4C (high suspicion for malignancy).

### Shear-Wave Elastography Data Acquisition and Analysis

The SWE probe was applied to the breast lesion, and kept still for a few seconds to allow adequate quality SWE images to be frozen and saved. The region-of interest (ROI) box of the color map included the entire lesion and the surrounding normal tissue, which was depicted on a semitransparent color map of tissue stiffness overlaid on the B-mode image with a range from dark blue, indicating the lowest stiffness, to red, indicating the highest stiffness (0–180 kPa). For quantitative measurement of SWE, fixed 2x2-mm sized ROI was placed by an investigator over the stiffest area of the lesion, including the immediate adjacent stiff tissue [19–26]. A second ROI of the same 2x2-mm sized ROI was placed in the normal breast fatty tissue. This allowed automatic calculation of quantitative parameters including maximum (Emax), mean (Emean), minimum (Emin) elasticity, standard deviation (SD), and the shear-wave elastography ratio (SWE-Ratio). The SWE-Ratio is between the mean elasticity of the mass and the mean elasticity of the adjacent adipose tissue.

### Core Needle Biopsy and Excision

All lesions underwent US-guided CNBs performed using a freehand technique with a 14-gauge CNB needle (Stericut with coaxial; TSK Laboratory, Tochigi, Japan) by one of four board-certified radiologists with 5–25 years of experience in US-guided breast biopsy. We obtained four to six core samples per lesion.

All lesions were excised after CNB. Of the 79 papillary lesions, 38 (48.1%) were surgically excised, and US-guided 8-gauge VAE (Mammotome Biopsy System, Ethicon Endosurgery, Cincinnati, OH, USA) was used for the remaining 41 lesions (51.9%). All atypical or malignant papillary lesions diagnosed at US-guided CNBs were surgically excised. Papillary lesions assessed as US category 4B or above were also surgically excised. US-guided VAE was performed by one of three radiologists with 5–7 years of experience in US-guided VAE. We used a high-resolution US unit (iu 22, Philips Healthcare, Bothell, WA, USA) for the US-guided VAE. Samples were taken by rotating the probe until the lesion was completely removed, as determined by real-time US. We obtained an immediate post-procedural US image to confirm completeness of the excision. Pathological reports obtained from the US-guided VAE or surgically excised tissues were regarded as final reference standards.

### Statistical Analysis

The independent two-sample *t*-test was used to compare continuous categorical variables between benign and atypical or malignant lesions. The chi-square or Fisher’s exact tests was used to compare categorical variables. Receiver operating characteristics (ROC) curves were prepared to evaluate SWE diagnostic performance. Optimal cutoff values for the quantitative parameters (Emax, Emean, Emin, SD, and SWE-Ratio) were calculated and used to obtain sensitivity and specificity. A *p*-value < 0.05 was considered as significant. All statistical analyses were performed using SPSS 21.0 (SPSS Inc., Chicago, IL, USA) and MedCalc ver. 15.6.1 (MedCalc Software, Mariakerke, Belgium) software.

## Results

### General Characteristics of the Breast Papillary Lesions

**Fig 1:**
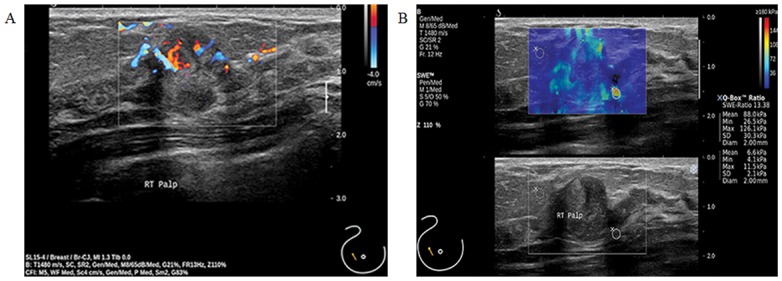
Atypical papilloma of the right breast in a 44-year-old woman. (A, B) This lesion was initially diagnosed a benign papilloma on ultrasound-guided core needle biopsy. This lesion was upgraded after surgical excision. (A) Color Doppler showing increased vascularity in internal portion of mass. (B) The ROI of shear wave elsatography image (top) is set at the margin of the lesion. B-mode ultrasound (bottom) image showing a 1.7-cm sized, irregular shaped and palpable breast lesion considered as category 4B. Adjacent ductal dilatation is also seen.

Of the 79 lesions, six (7.6%) were malignant and 12 (15.2%) were atypical. The upgrade rates to atypical papilloma and to malignancy were 8.9% (7/79) and 2.5% (2/79), respectively (Table 1, Fig 1). The final pathological diagnoses of the papillary lesions were intraductal papilloma (n = 61), intraductal papilloma with atypia (n = 12), intraductal papillary carcinoma (n = 4), and invasive papillary carcinoma (n = 2). Breast conserving surgeries were performed for all six malignant lesions.

**Table 1:** Summary of cases upgraded to atypical papilloma or malignancy after excision.

No.	Age (yr)	Size (mm)	Peripheral > 3cm	US cat.	Emax (kPa)	Emean (kPa)	Emin (kPa)	SD (kPa)	SWE-Ratio	Final pathology
1	48	7	No	4A	82.6	76.6	67.7	3.9	4.75	Atypical
2	47	5	No	4B	51.3	43.8	30.4	5.5	1.79	Atypical
3	44	17	No	4B	126.1	88	12.5	57.9	16.06	Atypical
4	50	9	Yes	4A	179	125.2	95.3	30.1	16.8	Atypical
5	46	15	Yes	4B	62.4	47.1	37.1	5.1	4.05	Atypical
6	77	30	Yes	4C	175.1	148.8	101.5	17.6	12.02	Malignancy
7	40	18	No	4C	121.3	102.4	80.7	11.4	9.06	Malignancy
8	21	12	No	4A	144.3	128.2	111	7.1	7.03	Atypical
9	48	10	No	4A	67.7	63.2	53.3	3.7	3.41	Atypical

Cat = category, Emax = maximum elasticity, Emean = mean elasticity, Emin = minimum elasticity, SD = standard deviation, SWE-Ratio = shear wave elastography-Ratio

Table 2 shows the general characteristics of the breast papillary lesions. personal history of breast cancer (*p* = 0.040), was significantly different between benign and atypical or malignant papillary lesions of the breast (Table 2). Peripheral location and adjacent ductal dilatation on US were also significantly different between benign and atypical or malignant papillary lesions of the breast (*p* < 0.05, Table 2). Atypical or malignant lesions (38.9%) were located periphery, comparing benign lesions (6.6%) (*p* < 0.001, Table 2). Adjacent ductal dilatations were more often seen in atypical or malignant lesions (50%) than those in benign lesions (11.7%) (*p* < 0.001, Table 2).

**Table 2:** General characteristics of the breast papillary lesions.

Variables	Benign (n = 61)	Atypical, Malignant (n = 18)	Total (n = 79)	*P* value
Clinical findings				
Age (years)*	47.5 ± 9.2	48.6 ± 13.8	47.7 ± 10.3	0.363
Previous breast cancer	2 (3.3%)	3 (16.7%)	5 (6.3%)	0.040
Palpability	10 (16.4%)	6 (33.3%)	16 (20.3%)	0.116
Nipple discharge	4 (6.6%)	3 (16.7%)	7 (8.9%)	0.185
Dense breast	49 (80.3%)	11 (61.1%)	60 (75.9%)	0.058
Ultrasound features				
Maximum size (mm)*	11.3 ± 5.7	13.2 ± 8.7	11.8 ± 6.5	0.119
Peripheral location	4 (6.6%)	7 (38.9%)	11 (13.9%)	<0.001
Breast thickness (mm)*	16.1 ± 4.0	19.6 ± 5.9	16.9 ± 4.7	0.071
Lesion depth (mm)*	9.5 ± 3.1	9.7 ± 2.8	9.5 ± 3.0	0.645
Duct dilation >3mm	7 (11.7%)	9 (50%)	16 (20.5%)	<0.001

* Mean ± standard deviations

### Conventional Ultrasound Findings

The US features of benign-and atypical or malignant papillary were evaluated. Non-parallel orientation, uncircumscribed margin, and internal vascularity were found in atypical and malignant lesions, indicating a significant pathological difference (*p* < 0.05). Vascularity was present in all malignant lesions. Parallel orientation (58/61, 95.1%), peripheral vascularity (42/61, 68.9%), and BI-RADS category 4A (47/61, 77.1%) were the most common findings in benign papillary lesions. Although the posterior feature was not significant (*p* = 0.929). Posterior enhancement was observed in 13.9% (11/79) of lesions.

US BI-RADS category was assessed as category 4A in 57 (72.2%, 47 in benign) lesions, category 4B in 16 (20.2%, 11 in benign), category 4C in five (6.3%, 2 in benign), category 5 in one (1.3%, 1 in benign) lesion. BI-RADS category was also significantly different according to the pathology (*p* = 0.002). Most of the benign (47/61, 77%) lesions were classified as BI-RADS category 4A. Most of the malignant lesions were classified as BI-RADS category 4C (3/6, 50%).

### Shear-Wave Elastography Findings with Diagnostic Performances

Table 3 summarizes the quantitative SWE parameters. All quantitative parameters were significantly different between benign and atypical or malignant lesions (p < 0.05, Table 3)

**Table 3:** Quantitative shear-wave elastography parameters.

Parameters	Benign	Atypical, Malignant	*P* value
Emax	73.68 ± 61.76 kPa	123.87 ± 65.00 kPa	0.004
Emean	61.05 ± 52.74 kPa	100.91 ± 42.22 kPa	0.005
Emin	49.07 ± 44.87 kPa	68.45 ± 29.96 kPa	0.09
SD	6.40 ± 5.27 kPa	15.22 ± 15.81kPa	<0.001
SWE-Ratio	5.27 ± 5.05	8.88 ± 6.88	0.017

Emax = maximum elasticity, Emean = mean elasticity, Emin = minimum elasticity, SD = standard deviation, SWE-Ratio = shear wave elastography-Ratio

**Fig 2:**
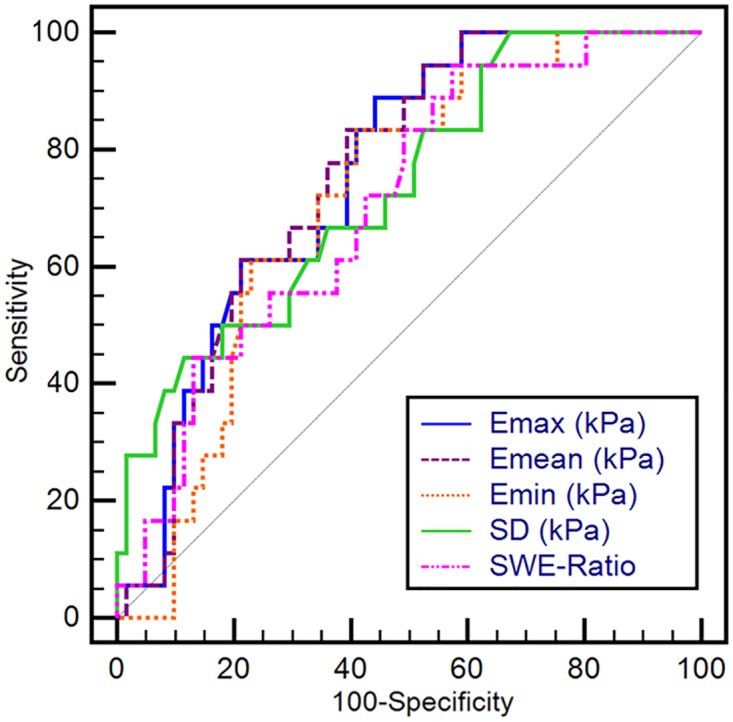
The receiver-operating characteristic curves (ROC) curves of shear-wave elastography for differentiating benign and atypical or malignant papillary lesions. The ROC curves for quantitative (Emax, Emean, Emin, SD, and SWE-Ratio) shear-wave elastography features were significantly different between benign and atypical or malignant papillary lesions (area under the curve, 0.707–0.757; sensitivity, 44.4–94.4%; specificity, 42.6–88.5%).

Table 4 and Fig 2 show the diagnostic performances of SWE for differentiating benign and atypical or malignant papillary lesions. Overall sensitivity and specificity of the SWE parameters were 44.4–94.4% and 42.6–88.5%, respectively (Table 4). Emax and Emean had the highest AUC value of 0.757 (95% confidence interval [CI], 0.648–0.847). The optimal Emax cutoff value was 62.1 kPa, with sensitivity of 88.9% and specificity of 55.7% (Table 4). When the optimal Emean cutoff value was 54.2 kPa, sensitivity was 83.3% and specificity was 60.7% (Table 4).

**Table 4:** Diagnostic performances of shear-wave elastography for differentiating between benign and atypical or malignant papillary lesions.

Parameters	Cutoff	Sensitivity (%)	Specificity (%)	AUC	95% CI
Emax	62.1 kPa	88.9	55.7	0.757	0.648–0.847
Emean	54.2 kPa	83.3	60.7	0.757	0.648–0.847
Emin	41.6 kPa	83.3	59	0.712	0.599–0.808
SD	11.9 kPa	44.4	88.5	0.732	0.621–0.826
SWE-Ratio	3.1	94.4	42.6	0.707	0.594–0.804

Emax = maximum elasticity, Emean = mean elasticity, Emin = minimum elasticity, SD = standard deviation, SWE-Ratio = shear wave elastography-Ratio

## Discussion

The treatment of atypical or malignant papillary lesions diagnosed at CNBs is surgical excision. Although the management of benign papillary lesions diagnosed at CNBs is still controversial [2–6], the current management of benign papillary lesions diagnosed at CNBs is US-guided VAE [4–7]. Thus, predicting atypical or malignant papillary lesions on US could be helpful for the management. Our result was similarly to that of previous studies [3, 8, 10,17], which reported irregular shape non-parallel orientation, uncircumscribed margin, internal vascularity, and high BI-RADS category. Compared to previous strain elastography results [17], SWE showed improved AUC and specificity for differentiating papillary lesions of the breast. In our study, the AUC of SWE was 0.707–0757. There are many studies on quantitative SWE demonstrating its useful diagnostic performance [21–26]. The quantitative SWE parameters, such as the maximum stiffness, mean stiffness SWE-Ratio, have improved diagnostic accuracy of breast ultrasound in previous studies [21–26]. In our study, the quantitative SWE parameters were significantly different in breast papillary lesions compared to those in other lesions.

US-guided CNBs diagnosed benign or atypical papillary lesions may be upgraded to atypical or malignant lesions after excision. Youk et al. [30] showed that lesion size ≥ 1 cm, lesions ≥ 3 cm from the nipple, and BI-RADS category are related to upgrading malignancies in patient ≥ 50 years of age. Our results showed that the upgrade rates to atypical papilloma and malignancy were 8.9% (7/79) and 2.5% (2/79), respectively (Table 1). In our study, 22.2% (2/9) of upgraded lesions were found in patient ≥ 50 years (Table 1). Six (66.7%) of nine upgraded lesions were ≥ 1 cm (Fig 1, Table 1), and three (33.2%) of nine upgraded lesions were in the periphery (>3 cm). Two lesions (22.2%) were category 4C and were finally proven to be malignancies. All upgraded lesions showed the optimal cutoff or above the quantitative SWE values, except one case (Table 1, No 2 case).

**Fig 3:**
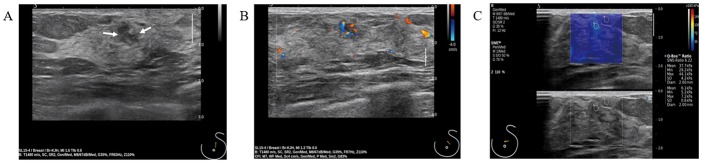
Benign intraductal papilloma diagnosed at ultrasound-guided core needle biopsy. (A-C) (A) B-mode ultrasound shows an irregular shaped hypoechoic mass (1 cm, arrows). (B) Color Doppler showing increased vascularity in the mass. This mass was assessed as category 4B. (C) Shear-wave elastography image showing a light blue color (Emax, 44.1 kPa). This mass was finally surgically excised and proved as benign intraductal papilloma.

Benign papillary lesions assessed as BI-RADS category 4A are generally enough to deal with US-guided VAE, whereas benign papillary lesions assessed as category 4C or 5 should be considered surgical excision in recent studies [4–7]. Regardless of BI-RADS assessment, atypical or malignant papillary lesions diagnosed at CNBs are recommended surgical excision [4–7]. All atypical or malignant papillary lesions initially diagnosed at CNBs were surgically excised in our study. Benign papillary lesions assessed as category 4B or above were also surgically excised. However, the management of the benign papillary lesions at CNBs with BI-RADS category 4B assessments is controversial. If our SWE cutoff value were applied to category 4B lesions, 3/16 (18.8%) category 4B lesions were downgraded into category 4A (Fig 3). These lesions were initially benign papillary lesions at CNBs, and finally proved as benign papillary lesions on final surgical excision. If SWE cutoff were applying to category 4B lesions, surgical excision could have been avoided. In our study, 11 benign papillary lesions were assessed as category 4B.

If applying our cutoff value to benign papillary lesions assessed as category 4B, 1/5 (20%) lesions were false-negative results and 3/11 (27.2%) lesions were false-positive results. Although high false-positive rate (27.2%), low false-negative rate (20%) might prevent misdiagnosis or underestimation of malignant papillary lesions. Likewise, our results, Yoon et al. [22] reported that false-positive rates were significantly higher than false-negative rates in SWE. Large size, breast thickness, depth and fair quality influence false-positive rates in SWE [22]. To reduce false-positive rates, additional clinical findings should be considered with conventional US and SWE findings. There was one false-negative case in our study (Table 1, No 2 case). The size of this lesion was 5 mm. As similarly, small size influences false-negative results [22, 31, 32]. Small invasive cancers (≤ 10mm) are frequently showed soft elasticity [31]. Chang et al. [32] reported the mean stiffness of cancers up to 5mm was 86.8 kPa and 112 kPa for the mean elasticity of 6–10 mm-sized cancers. Thus, small size may lead a false-negative result in our study.

**Fig 4:**
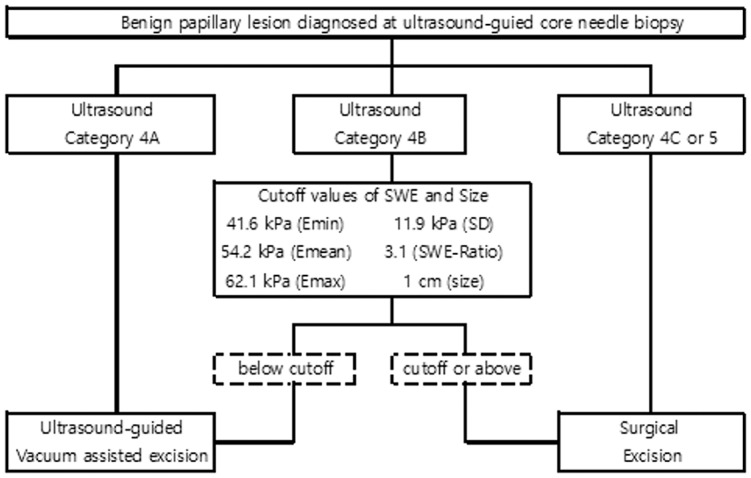
Management for the benign papillary lesions diagnosed at ultrasound-guided core needle biopsy.

In this study, US-guided VAE was performed in 51.9% (41/79) of papillary lesions. All 41 lesions were diagnosed initially as benign using the CNB and none were upgraded after US-guided VAE. All lesions managed with US-guided VAE were category 4A in our study. In our study, two benign lesions managed with US-guided VAE were > 1.5 cm (1.7 and 1.9 cm). However, these lesions were category 4A and no residual or newly developed lesions were detected on 1-year follow-up images. In accordance with our results and previous studies [4–7, 30], we may suggest the guideline for the benign papillary lesions diagnosed at CNBs (Fig 4). Papillary lesions more than 1 cm is recommended surgical excision rather than US-guided VAE.

There were several limitations in our study. The first limitation was the small number of cases. Of 217 papillary lesions diagnosed by US-guided CNB, only 79 (36.4%) were finally included due to the lack of a SWE image or no excision. Thus, large numbers and multicenter studies are necessary in the future. Second, we did not compare our results with those of conventional US or assess combined diagnostic performance. Youk et al. [33] reported that adding SWE features to BI-RADS improves diagnostic performance and may be helpful to stratify category 4 lesions. However, we focused on the SWE parameters to differentiate papillary lesions. Further studies combining SWE and conventional US in are necessary to differentiate papillary lesions. Third, we used a 2x2-mm sized ROI. Larger ROI might be more accurate for the assessment of the breast masses by providing both maximum stiffness and heterogeneity of breast lesions. However, many previous studies used a 2x2-mm sized ROI for quantitative SWE analysis [19–26]. Although 2x2-mm sized ROI is widely used, the ROI size can affect the diagnostic performance of SWE [34]. The benign/malignant threshold was lower with increasing ROI size [34]. Skerl et al. [34] also suggested that large ROI sizes reflected the heterogeneity of malignant tissue. Using ROC analysis, the effect of the ROI size was clearly verified for Emean and SD [34]. Especially, Emean represented the mean of all SWE parameters within the ROI [34]. Although we used 2x2-mm sized ROI, Emean and Emax showed the highest AUC in our results. However, further study about breast papillary lesions using larger ROI size may be necessary.

In conclusion, SWE provided additional information for the differential diagnosis of breast papillary lesions. Quantitative SWE features helped differentiate papillary lesions of the breast.
